# Trigonelline reverses high glucose-induced proliferation, fibrosis of mesangial cells via modulation of Wnt signaling pathway

**DOI:** 10.1186/s13098-022-00798-w

**Published:** 2022-02-09

**Authors:** Chen Chen, Yan Shi, Jiulong Ma, Zhen Chen, Ming Zhang, Yan Zhao

**Affiliations:** 1 College of Pharmacy, Jilin University, Xinmin Street No. 1163, Changchun, People’s Republic of China; 2 Physical Examination Center, Jilin University Second Hospital, Street No. 218, Changchun, Ziqiang People’s Republic of China

**Keywords:** Diabetic nephropathy, Trigonelline, Mesangial cells, Wnt/β-catenin, Apoptosis

## Abstract

**Background:**

Diabetic nephropathy (DN) is the leading cause of the end-stage renal disease (ESRD). The proliferation and apoptosis of mesangial cells induced by the activated Wnt/β-catenin pathway is crucial in DN. Trigonelline (TRL) is an alkaloid that has been shown to decrease proteinuria and protect the renal function in DN. However, the effect of TRL on the Wnt/β-catenin pathway of mesangial cells is unclear.

**Methods:**

As a cellular DN model, human mesangial cells (HMCs) were treated with high-glucose (HG). β-Catenin plasmid and control knockdown plasmids were transfected into HG-treated HMCs as β-catenin pcDNA and β-catenin siRNA groups, respectively. Cell viability was measured by MTT assay. Flow cytometry was used to detect the cell cycle. Cell apoptosis was evaluated by flow cytometry and terminal dUTP transferase nick end labeling (TUNEL) assay. mRNA expression of Wnt1, Wnt3a, Wnt4, Wnt5a, β-catenin, TCF4, Cyclin D1, and CDK4 were detected by qRT-PCR. Protein expression of Wnt4, Wnt5a, nucleus-β-catenin, TCF4, Cyclin D1, and CDK4 were detected by western blotting.

**Results:**

TRL significantly inhibited HG-induced HMCs viability over three-time points measured (24, 48, and 72 h). In addition, TRL suppressed the levels of fibronectin (FN) and collagen IV (Col IV) in HG-stimulated HMCs. Furthermore, TRL efficiently inhibited the activation of the Wnt/β-catenin signaling pathway in HG-stimulated HMCs. Taken together, these data indicated that TRL inhibited HG-induced HMCs proliferation and ECM expression via the modulation of the Wnt signaling pathway.

**Conclusions:**

TRL reduces HG-induced cell injury by regulating the Wnt/β-catenin signaling pathway.

## Introduction

Diabetic nephropathy (DN) has become the third-largest threat to human health after cancer [1]. There is no specific treatment for DN; the primary therapy combines hypoglycemic drugs and angiotensin receptor blockers or angiotensin-converting enzyme inhibitors. However, this therapy has disadvantages, including high costs, many side effects, and a long course of treatment [2–4]. Therefore, exploring the pathogenesis of DN and finding effective anti-DN drugs are of great importance. DN pathological changes include thickening of the basement membrane, accumulation of extracellular matrix, diffuse glomerular sclerosis, the most severe development of renal failure [5–7]. The extracellular matrix is the main pathological change of mesangial cell proliferation. The accumulation of extracellular matrix and hypertrophy of mesangial cells eventually leads to glomerular sclerosis [8]. Therefore, the function of mesangial cells plays an essential part in the clinical diagnosis and treatment of DN. Therefore, the function of mesangial cells plays an essential part in DN's clinical diagnosis and treatment. Kim, Jardim, Yang, and others have confirmed that chronic hyperglycemia promotes the molecular pathway leading to apoptosis and causes glomerular damage [9–11]. Therefore, hyperglycemia-induced excessive proliferation and apoptosis appear to be crucial factors in the progression of glomerulosclerosis.

The Wnt/β-catenin signaling pathway comprises Wnt ligand–protein outside the cell, receptors on the cell membrane, signaling part in the cytoplasm, and transcription regulation part in the nucleus [12]. The main components of this pathway include Wnt protein family, Frizzled/low-density lipoprotein receptor-related protein (Fz/LRP), Dishevelled protein (Dsh) in the cytoplasm, β-catenin Protein (β-catenin), glycogen synthase kinase-3 (GSK-3), scaffold protein (axin/conductin), adenomatous polyposis coli (APC) and T cells T cell factor/lymphoid enhancer factor (TCF/LEF) transcription factor family [13–15]. In the absence of Wnt ligands, the concentration of β-catenin is controlled by the Axin/GSK-3β/APC complex, which phosphorylates β-catenin, leading to the degradation of β-catenin in the cytoplasm. When the Wnt signal exists, the Axin/GSK-3/APC complex depolymerizes, the β-catenin in the cytoplasm can be stabilized and accumulated, enter the nucleus and combine with TCF to initiate downstream gene transcription. Wnt protein expression is a vital initiation signal for the activation of the Wnt/β-catenin signaling pathway. At present, it is known that 19 types of Wnt proteins are mainly involved. Wnt1, Wnt3, Wnt3a, Wnt5a, Wnt7b, Wnt8 are involved in the Wnt classic signaling pathway. The Wnt/β-catenin pathway plays an essential part in various kidney diseases, including DN [16, 17].

*Trigonella foenum-graecum* L. (fenugreek), a traditional Chinese herb, and its components are beneficial in preventing and treating diabetes and central nervous system disease. Trigonelline (TRL) is an active component in the extract of fenugreek seeds. Trigonelline has hypoglycemic, hypolipidemic, neuroprotective, and it has been shown to alleviate kidney damage in STZ-diabetic rats [18, 19]. Whether TRL affects glomerular mesangial cells in HG-stimulated and is mediated by Wnt/β-catenin is not clear at present. In this study, we investigated the effects of TRL on mesangial cell proliferation, Wnt/β-catenin pathway signaling in response to high glucose.

## Materials and methods

### Cell culture

The American Type Culture Collection provided HMCs. The cells were cultured in 5.5 mm d-glucose (10% thermally inactivated fetal bovine serum) and placed in a 37 °C, 5% CO_2_ incubator. The cells were sub cultured and every two days and divided into seven groups. Group NG was treated with normal glucose (NG, 5.5 mM), group HG was treated with high glucose (HG, 30 mM), group A was treated with a medium dose of TRL (100 μM, wkq-00259, purity > 98%; Vic’s Biological Technology Co. Ltd., Sichuan, China), group B was transfected with β-catenin siRNA and then treated with TRL (100 μM), group C was transfected with β-catenin pcDNA, group D was transfected with β-catenin pcDNA then treated with TRL (100 μM), and group E was treated with the Wnt/β-catenin signaling pathway inhibitor ICG-001 (3 µM; HY-14428, MedChemExpress Co. Ltd., New Jersey, USA), group A, B, C, D, E groups were incubated in high glucose containing media.

### Cell viability assay

Exponentially growing cells (3 × 10^3^/well) were plated in 96-well plates, serum-starved overnight and grouped by NG and HG. Then divided into individual TRL concentration administration groups (0, 25, 50, 100, 200, 400, and 800 µM) for different time durations (24, 48, and 72 h). 24.5 mM mannitol (MA) was added to the NG medium as an osmotic control. After 24 h, 48 h and 72 h, 100 µL MTT (0.5 mg/mL, Sigma-Aldrich) was added to each well. After 4 h, 150 μL DMSO (Sigma-Aldrich) was added to each well. The absorbance of each well was measured using a microplate reader (SpectraMax Plus384; Molecular Devices, USA) with a reference wavelength of 490 nm.

### Immunocytochemistry analysis

HMCs were used as monolayer cultures and fixed in 4% paraformaldehyde for 10–15 min. After the cells were blocked with an endogenous peroxidase blocker (ZSGB-BIO, Beijing, China) for 20 min. Incubation with rabbit monoclonal antibodies against β-catenin (ab32572, 1:200; Abcam, USA) at 4 °C overnight. Then, the bound antibody was detected with horseradish peroxidase (HRP)-anti-rabbit IgG and diaminobenzidine (DAB) and then counterstained with hematoxylin. The percentage of positively stained areas in the glomeruli was analyzed by computer image.

### Transient transfection

HMC was cultured in groups on a six-well plate. After the cells reached approximately 80% confluence, 2.5 µg of plasmids expressing CTNNB1 (HG11279-M-F, Sino Biological Inc., Beijing, China) were transfected into the HMCs to overexpress β-catenin using Lipofectamine 2000 reagent (11668-027, Invitrogen-BRL, Carlsbad, CA, USA) following the manufacturer’s protocol. For β-catenin knockdown, 75 pmol of small interfering RNA (siRNA) against CTNNB1 (GenePharma Corporation, Shanghai, China) was transfected into the HMCs using Lipofectamine 2000 reagent. The siRNA sequences are as follows: si-CTNNB1 (Human) sense 5′-GGGUUCAGAUGAUAUAAAUTT-3′ and antisense 5′-AUUAUAUCAUCUGAACCCAG-3′. The siRNA/liposome and pcDNA/liposome complexes were added to the serum-free medium and the complexes were formed at room temperature for 20 min. After 4 h of incubation, the medium containing HG (30 mM) was added and the pregnancy continued. After 48 h of incubation, cells were harvested to detect β-catenin expression and related targets.

### PCR analysis

TRIzol Reagent (15596026, Invitrogen, USA). PCR was executed on the 7500 FAST Real-Time PCR System for 45 cycles (Applied Biosystems, Carlsbad, CA). The thermocycling conditions were as follows: initial melting at 94 °C for 30 s followed by 45 cycles of 94 °C for 5 s, 51 °C for 15 s, and 72 °C The 10% sec. The GAPDH mRNA was amplified simultaneously and used as a loading control. The relative amounts of mRNA were determined by the 2^−ΔΔCt^ method. Each sample was tested in triplicate. Primer sequences are shown in Table 1.

**Table 1:** Sequences of specific primers for qRT-PCR

Genes	Primers	Product length (bp)
CTNNB1	Forward: 5′-ACAGGGAAGACATCACTGAGCCTGC-3′	211
	Reverse: 5′-GGTGCATGATTTGCGGGACAAAG-3′	
Wnt 1	Forward: 5′-TCCCCTTTGTCCTGCGTTTT-3′	207
	Reverse: 5′-CTGGGAGAATGGGGGCATTT-3′	
Wnt 3	Forward: 5′-AGGACAAGTATGACAGCGCC-3′	164
	Reverse: 5′-CTCTGGGTTGGGCTCACAAA-3′	
Wnt 4	Forward: 5′-ATGAGTCCCCGCTCGTG-3′	242
	Reverse: 5′-TGGTACTGGCACTCCTCAATG-3′	
Wnt5a	Forward: 5′-TCCATTCCTGGGCGCATC-3′	289
	Reverse: 5′-CCATCCCCAAAGCAACTCCT-3′	
TCF4	Forward: 5′-CCAATCACGACAGGAGGATT-3′	298
	Reverse: 5′-CGACCTTTGCTCTCATTTCC-3′	
Cyclin D1	Forward: 5′-GCTGCGAAGTGGAAACCATC-3′	135
	Reverse: 5′-CCTCCTTCTGCACACATTTGAA-3′	
CDK4	Forward: 5′-ATGGCTACCTCTCGATATGAGC-3′	124
	Reverse: 5′-CATTGGGGACTCTCACACTCT-3′	
GAPDH	Forward: 5′-CAAGGTCATCCATGACAACTTTG-3′	496
	Reverse: 5′-GTCCACCACCCTGTTGCTGTAG-3′	

### Western blot analysis

Total Protein Extraction Kit (SD-001, Invent Biotechnologies Inc., USA). For separation, an equal amount of protein was loaded onto a 12% sodium dodecyl sulfate–polyacrylamide gel. The protein was then transferred to a polyvinylidene fluoride membrane (Merck Millipore, MA, USA) and blocked with 5% skim milk for 1 h. Incubate the membrane and primary antibodies overnight at 4 °C. The next day, HRP-conjugated anti-rabbit IgG secondary antibody (ZB-2301, 1:3000; ZSGB-Bio) was incubated for 1 h. Images were captured using the Tanon 4200 Automatic Chemiluminescence Image Analysis System (Tanon, Shanghai, China) and analyzed using Image-Pro Plus 6.0.

### Cell cycle

The HMC was inoculated to a 6-well plate with a density of 1 × 104. After 48 h, PBS was washed twice and collected, fixed in 70% cold ethanol at 4 °C overnight. Next, the fixed cells were resuspended at room temperature with 50 g/mL Propidium iodide (PI). (CF0031, Beijing dingguo biotechnology co., LTD.) binding buffer in 30 min. Then flow cytometry for detection. (Becton Dickinson, SAN Jose, ca, USA) ModFit LT V3.3.11 (Verity Software House Inc., topsom, Maine, USA) was used to determine the proportion of G0/G1, S and G2/M cells.

### Cell apoptosis analysis

Annexin v-FITC/PI kit detected cell apoptosis. (ca1020-50, Solarbio Science & Technology Co. Ltd., Beijing, China) Cells were washed with pre-cooled PBS and collected, then fixed at 4 °C overnight in 70% ethanol. After 72 h of incubation, cells were collected and centrifuged at 4 °C and 100×*g* for 5 min; gently aspirate the supernatant and mix well with 1 mL PBS. Annexin V—FITC and PI (5 μL each) were then added to tubes, mixed gently, and incubated for 15 min. Then, 300 mL of binding buffer was added and apoptosis was detected within 1 h.

### TUNEL analysis

The TUNEL apoptosis detection kit was used (C1089, Beyotime Institute of Biotechnology, Shanghai, China). DNA strand breakage was evaluated by fluorescence labeling of the terminal dUTP notch (TUNEL). DAPI solution (c02-04002, Bioss Biotechnology Co. Ltd., Beijing, China) used nuclear staining. TUNEL positive cells were counted in the defined area and their density was calculated.

### Statistical analysis

Data are presented as mean ± SEM with n denoting the sample size in each group. Differences between groups were analyzed by variance analysis or rank sum test. One-way ANOVA tests were used to compare three or more independent groups. SPSS software was used to test the normality of the data and statistical analysis (SPSS, Armonk). We considered a p-value < 0.05 as statistically significant.

## Results

### TRL suppressed HG-induced excessive proliferation of HMCs

**Fig. 1:**
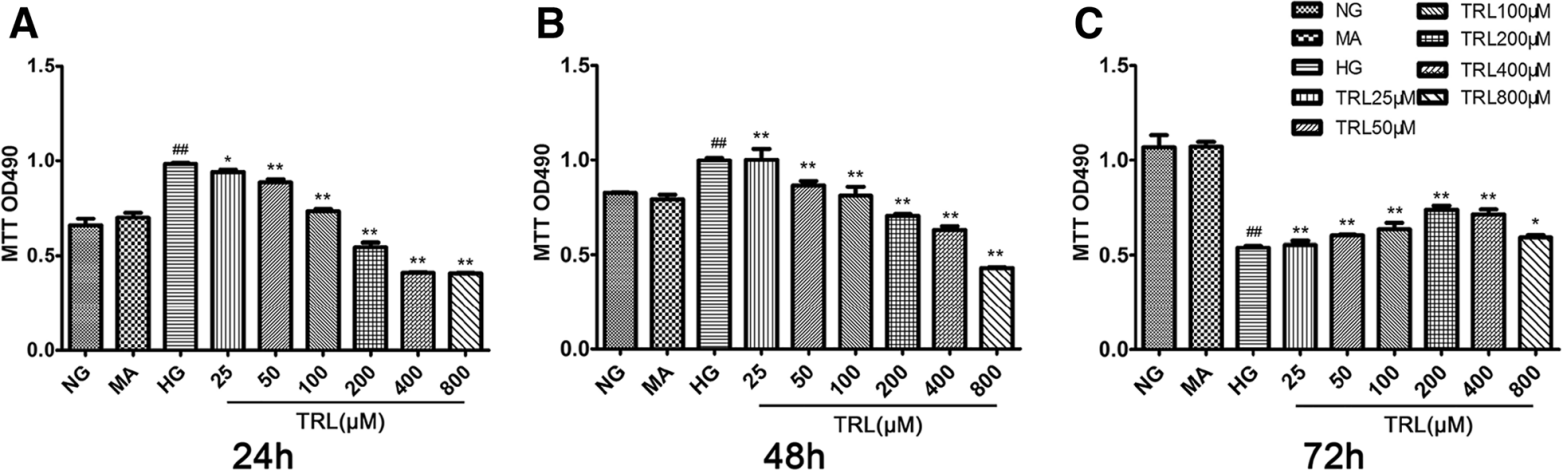
Growth-inhibitory effects of TRL on HMCs under the high-glucose conditions. Cell viability was measured by an MTT assay. Cells were treated with various concentrations of TRL (25–800 μM) for 24 h (**A**), 48 h (**B**), and 72 h (**C**); mannitol (24.5 mM, MA) was used as an osmotic control. The data are presented as the mean ± SEM from at least three independent experiments. *NG* normal glucose, *HG* high glucose, *MA* mannitol, TRL (25–800 μM): TRL + high glucose; ^#^P < 0.05 versus NG, ^##^P < 0.01 versus NG; *P < 0.05 versus HG, **P < 0.01 versus HG

To examine the effect of TRL on HG-induced HMCs, HMCs were cultured and the MTT assay was used to detect cell proliferation for different periods (24, 48, and 72 h). Cells cultured under HG conditions for 24 h increased faster than the untreated cells (P < 0.01), and the difference was still significant after 48 h (P < 0.01). However, the proliferation rate of HG-treated cells decreased after 72 h. Treatment with mannitol did not alter cell growth at any time tested, suggesting that the HG-induced HMC proliferation was not due to high osmotic pressure. Compared to the proliferation rate in the HG group, that in the cells treated with TRL at a concentration of 25 to 800 μM decreased significantly (P < 0.05), and the effect was concentration-dependent (Fig. 1). The results show that HG can effectively promote the proliferation of HMCs in a time-dependent manner, and this effect was most significant at 48 h. TRL suppressed the HG-induced excessive proliferation of HMCs in concentration- and time-dependent methods for the first 48 h. Therefore, we chose the 48 h time point for further experiments, including cell-cycle, western blot, qRT-PCR, and immunocytochemistry analyses. We chose the 72 h time point for the apoptotic analyses.

### TRL inhibits ECM accumulation in HMCs

**Fig. 2:**
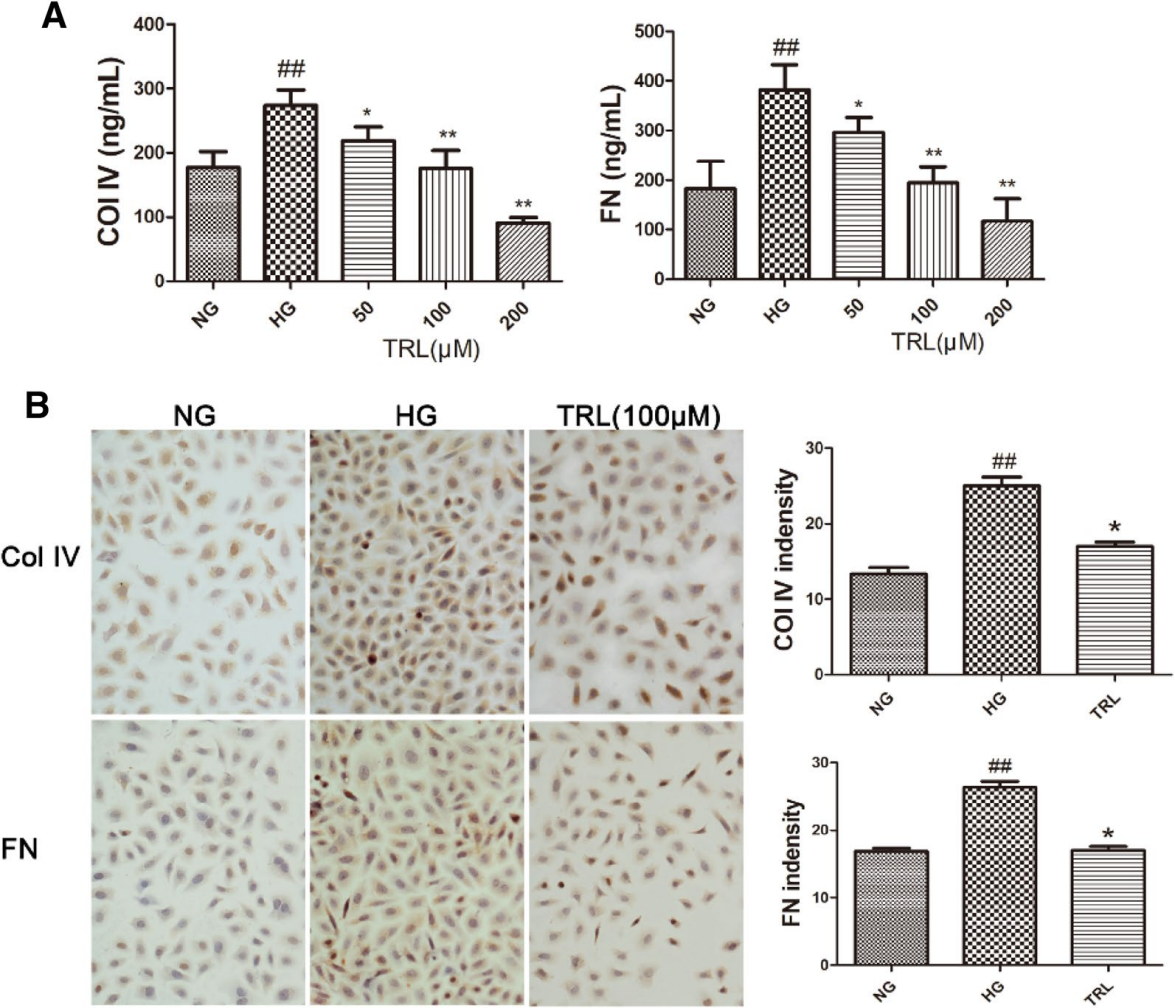
Expression of ECM accumulation in HMCs under the high-glucose conditions. **A** Elisa method detects Col IV and FN. **B** Immunohistochemical staining (×200) of Col IV and FN. *NG* normal glucose, *HG* high glucose, TRL (50, 100, 200 μM): TRL + high glucose; ^#^P < 0.05 versus NG, ^##^P < 0.01 versus NG; *P < 0.05 versus HG, **P < 0.01 versus HG

Elisa's results showed that compared with the NG group, HG treatment increased the expression levels of ECM components fibronectin and collagen IV; compared with the HG group, the secretion levels of Col IV after TRL treatment FN of cells were significantly reduced (Fig. 2A). Continue to detect the expression of Col IV and FN by immunocytochemistry, and the results are consistent with the results of Elisa (Fig. 2B). Compared with the NG group, the brown staining of Col IV and FN cells in the HG group was significantly more profound, and the expression increased significantly; after 100 μM TRL intervention, the brown staining of HMCs decreased, and the expression of FN and Col IV decreased significantly.

### TRL suppresses the expression of various wnts in HMCs

**Fig.3:**
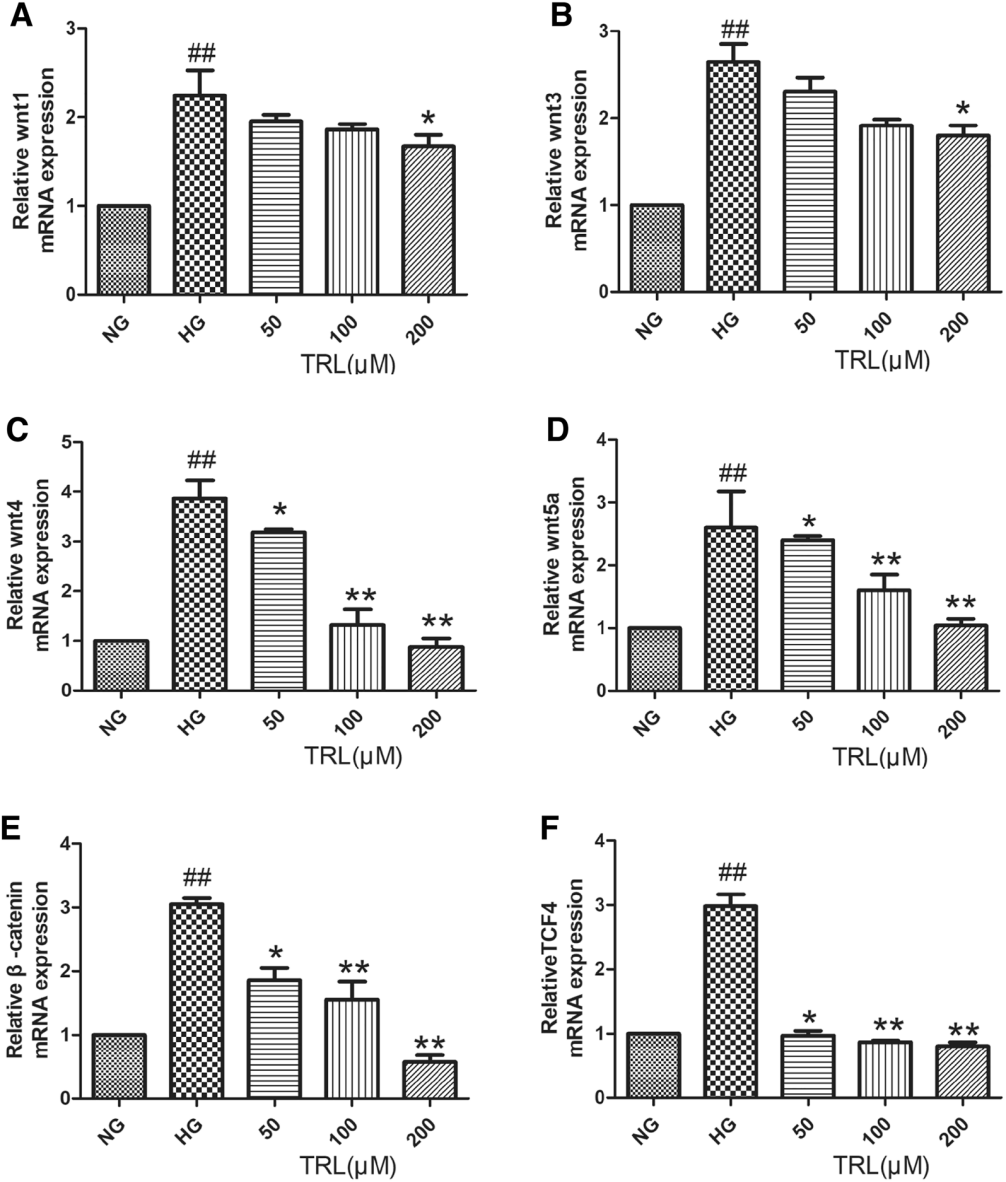
Activation of the Wnt/β-catenin pathway induced by high glucose in HMCs. **A**–**F** The relative Wnt1, Wnt3, Wnt4, and Wnt5a, β-catenin, and TCF4 mRNA expression detected by qRT-PCR. Total RNA was extracted from treated cells and assessed by qRT-PCR analysis. *NG* normal glucose, *HG* high glucose, TRL (50, 100, 200 μM): TRL + high glucose; ^#^P < 0.05 versus NG, ^##^P < 0.01 versus NG; *P < 0.05 versus HG, **P < 0.01 versus HG.

Since the Wnt/β-catenin pathway plays an essential role in DN development [12], the present study investigated the Wnt/β-catenin pathway in HMCs exposed to HG. As indicated in Fig. 3A–D, HG treatment significantly induced the expression of a variety of Wnts in HMCs, including Wnt1, Wnt3, Wnt4, and Wnt5a, compared with the NG group. However, the mRNA expression of wnt4 and wnt5a were significantly decreased. After the effect of TRL, wnt4 and wnt5a were significantly down-regulated, while Wnt1 and Wnt3 did not change significantly. It seems that TRL has a significant effect on Wnt4 and Wnt5a. To examine the biological consequence of Wnt induction in HG- induced HMCs injury, the mRNA expression of β-catenin and TCF4 were also investigated in HMCs treated with HG. As shown in Fig. 3E, F, the RT-PCR approach revealed a marked increase of β-catenin mRNA expression in HMCs by 48 h after HG treatment. The above reaction was reversed after TRL action, the mRNA expression of β-catenin and TCF4 were markedly decreased. These results suggested that the canonical Wnt pathway might play a key role in HG-treated HMCs.

### TRL down-regulated Wnt/β-catenin signaling in HMCs under HG conditions

**Fig. 4:**
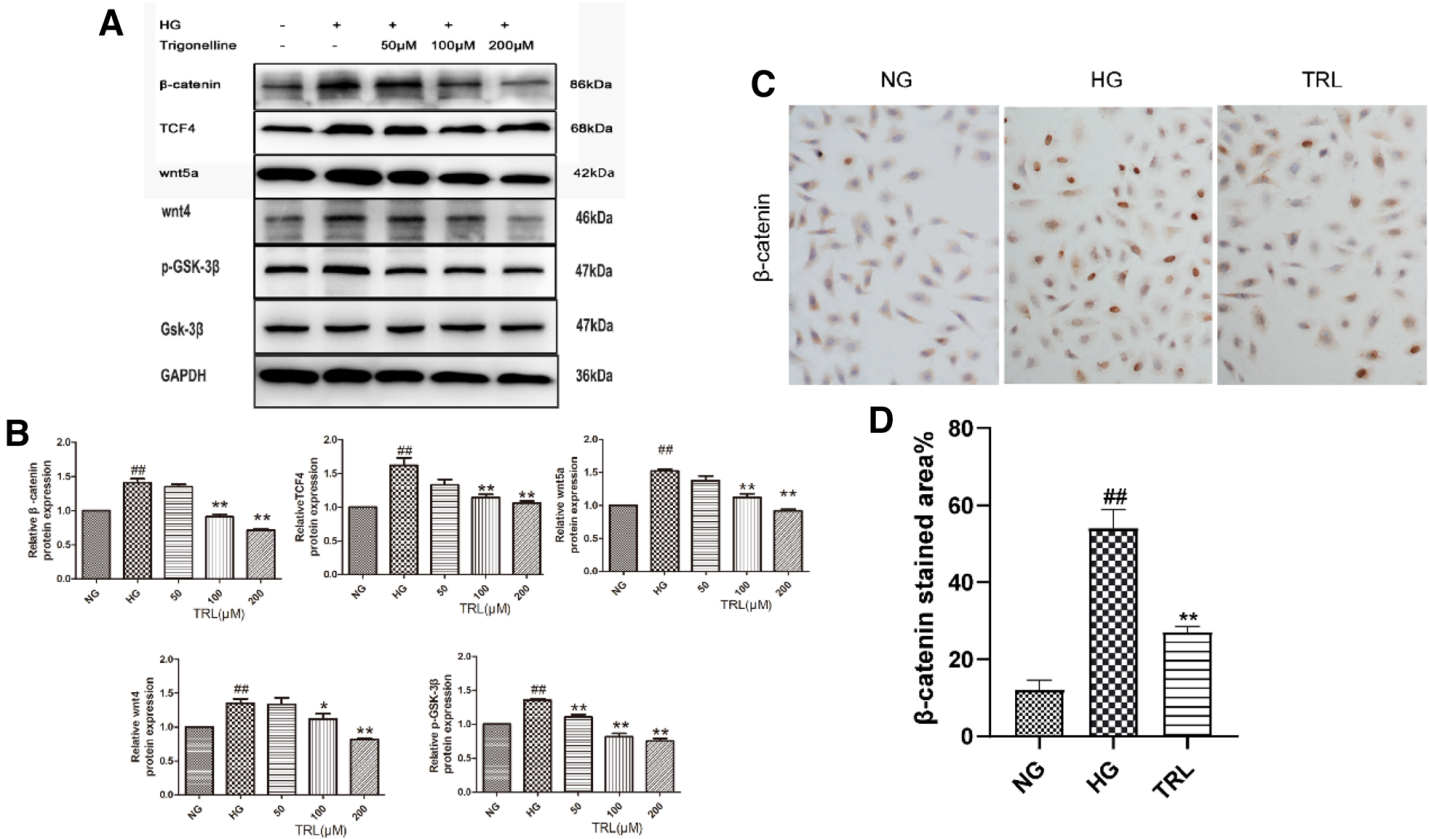
Effects of TRL on β-catenin, Wnt5a, and TCF4 expression in HMCs. HMCs were treated with various concentrations of TRL (50, 100, or 200 μM) for 24 h. Proteins were extracted from cells and assessed by western blot analysis. Total RNA was extracted from treated cells and assessed by qRT-PCR analysis. GAPDH served as the loading control. **A** Representative western blots of β-catenin, TCF4, Wnt5a, Wnt4, and GSK-3β expression. **B** β-catenin, TCF4, Wnt5a, Wnt4, and GSK-3β protein expression were detected by western blot. **C** Immunohistochemical staining (×200) of β-catenin, Wnt5a, and TCF4. *NG* normal glucose, *HG* high glucose, TRL (50, 100, 200 μM): TRL + high glucose; ^#^P < 0.05 versus NG, ^##^P < 0.01 versus NG; *P < 0.05 versus HG, **P < 0.01 versus HG

We tested three concentrations of TRL (50, 100, and 200 μM) for these experiments to choose the optimum concentration. As shown in Fig. 4A, B, the protein expression of nucleus-β-catenin, TCF4, Wnt5a, Wnt4, and GSK-3β were significantly increased following HG treatment; TRL reversed the increase in the expression of nucleus-β-catenin, Wnt4, Wnt5a, TCF4, and GSK-3β, with the most significant response observed at the 100 μM concentration. Therefore, we used 100 μM TRL in all subsequent experiments. The immunohistochemistry staining results show that the expression of β-catenin is higher in the HG group than that in the NG group. Of these, the staining for β-catenin was positive. We observed that HG promoted β-catenin entry into the nucleus. Compared to the HG group, the number of positive cells in the TRL-treated group significantly decreased (Fig. 4C, D). These results suggest that TRL inhibited the HG-induced HMC proliferation by inhibiting the Wnt/β-catenin signaling pathway.

### Signaling mechanism for the anti-diabetic property of TRL in HMCs

The above results demonstrate the beneficial effects of TRL against HG-induced over-proliferation of HMCs. To shed light on how TRL mediates these effects, we use the Wnt/β-catenin pathway inhibitor ICG-001 and study the expression of nucleus-β-catenin, Wnt5a, and TCF4. We found that TRL had the same impact on these signals as ICG-001. These results indicate that TRL might influence the survival of HMCs by inhibiting the Wnt pathway. Next, we chose to transfect HMCs with plasmids expressing β-catenin transiently. We found that the signals of the Wnt/β-catenin pathway increased significantly but decreased when treated with TRL at the same time. These results revealed that HG might lead to an increase in β-catenin.

**Fig. 5:**
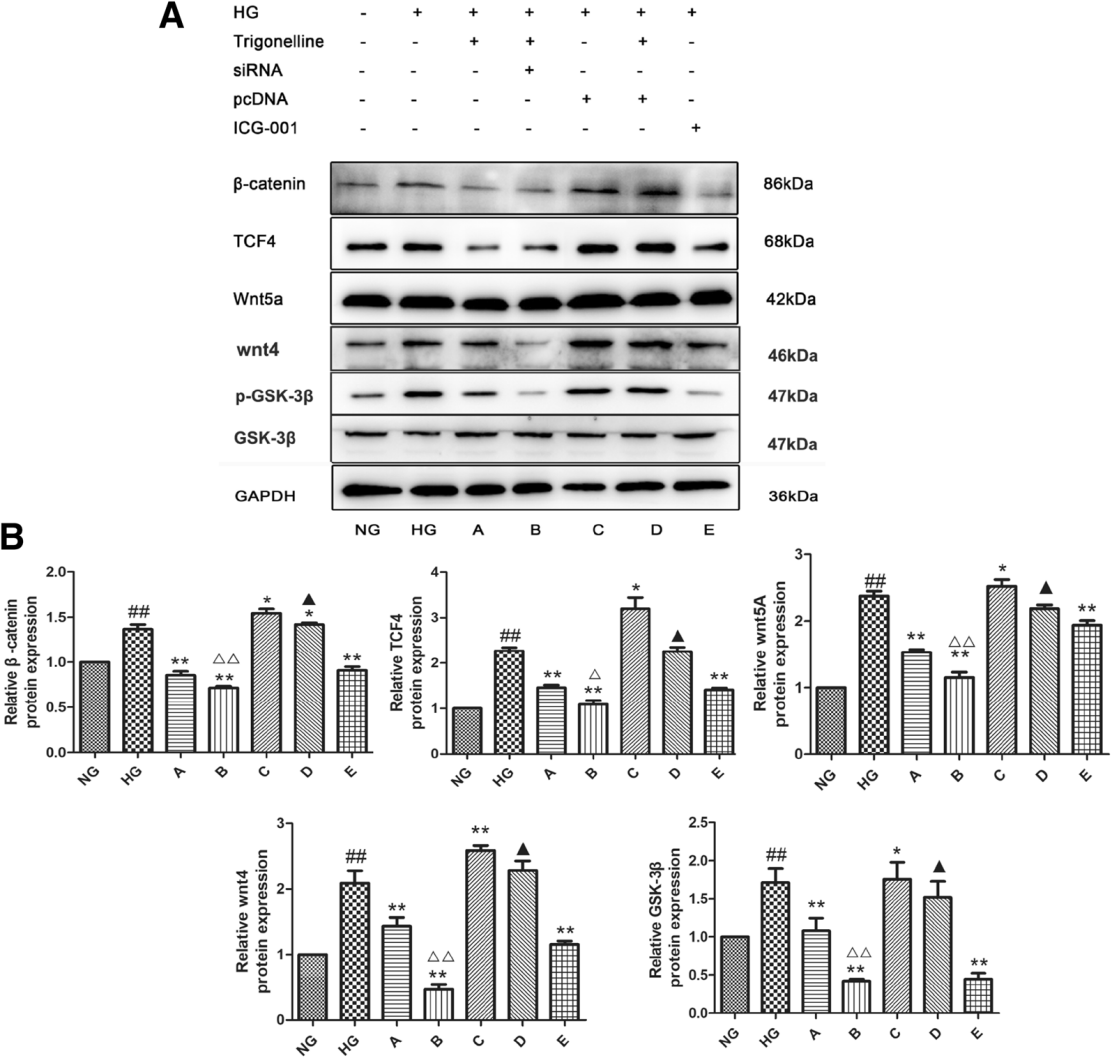
Expression of β-catenin, TCF4, Wnt5a, Wnt4, and GSK-3β in HMCs. After treatment, proteins were extracted from the cells and assessed by western blot analysis. GAPDH served as the loading control. **A** Representative western blot of β-catenin, TCF4, Wnt5a, Wnt4, and GSK-3β expression. **B** The relative β-catenin, TCF4, and Wnt5a protein expression detected by western blot. *NG* normal glucose, *HG* high glucose, **A** TRL + high glucose, **B** TRL + β-catenin siRNA + high glucose, **C** β-catenin pcDNA + high glucose, **D** TRL + β-catenin pcDNA + high glucose, **E** ICG-001 + high glucose; ^#^P < 0.05 versus NG, ^##^P < 0.01 versus NG; *P < 0.05 versus HG, **P < 0.01 versus HG; ^△^P < 0.01 versus group A, ^△△^P < 0.01 versus group A; ^▲^P < 0.05 versus group C, ^▲▲^P < 0.01 versus group C

Additionally, TRL reduced the upregulation of the mRNA and protein levels of β-catenin. Compared with that observed for the HG group, the expression of each signal factor was significantly down-regulated in the 100 μM of TRL and the β-catenin siRNA group (group B; P < 0.05), and the expression level was also significantly reduced compared to that of group A treated with TRL alone (Fig. 5). These results indicate that TRL mediates when the Wnt signaling is active. TRL not only down-regulates the high expression of β-catenin but also has a significant regulatory impact on the other factors in the Wnt/β-catenin signaling pathway.

### TRL suppressed HG-induced cell-cycle progression in HMCs

**Fig. 6:**
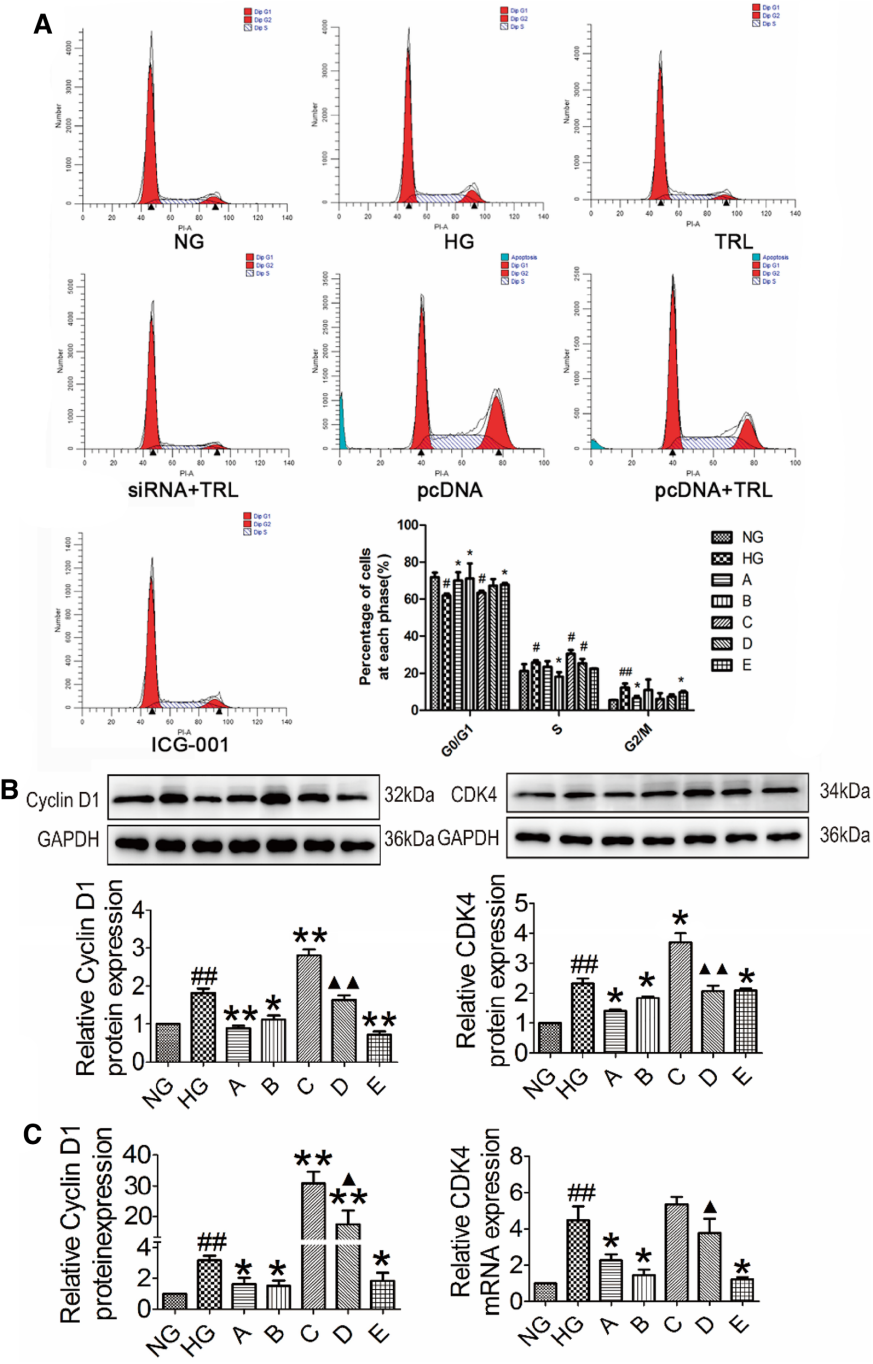
Effects of TRL on the cell cycle of HMCs induced by high glucose (**A**). Effects of TRL on Cyclin D1 and CDK4 protein and mRNA expression in HMCs (**B**, **C**). The cell cycle distribution was determined by assessing the individual nuclear DNA content reflected by the fluorescence intensity of incorporated PI. Flow cytometry analyzed the percentages of cells in G0/G1, S, and G2/M phases after the indicated treatments. Representative flow cytometry graphs are shown and the results are presented as the mean ± SEM from at least three independent experiments. *NG* normal glucose, *HG* high glucose, **A** TRL + high glucose, **B** TRL + β-catenin siRNA + high glucose, **C** β-catenin pcDNA + high glucose, **D** TRL + β-catenin pcDNA + high glucose, **E** ICG-001 + high glucose; ^#^P < 0.05 versus NG, ^##^P < 0.01 versus NG; *P < 0.05 versus HG, **P < 0.01 versus HG, ^△^P < 0.05 versus A, ^△△^P < 0.01 versus A; ^▲^P < 0.05 versus C, ^▲▲^P < 0.01 versus C

Flow cytometry analysis was performed further to examine the role of TRL in HMC cell-cycle progression. As shown in Fig. 6A, HG-induced a decrease in the percentage of cells in the G1 phase but increased those in the S phase, indicating that HG promoted cell cycle progression. In contrast, TRL arrested cells in the G1 phase (p < 0.05), and therefore, the number of cells in the S phase was significantly lower (p < 0.05). After treatment with siRNA and TRL (group B), the lowest proportion of cells was in the S phase. The ICG-001 intervention (group E) also had a concrete block in the S phase. The acceleration of the cell cycle in the β-catenin overexpression group (group C) was more significant than that in the HG group, and the percentage of cells in the S phase for group D was significantly reduced after treatment with TRL (P < 0.05). High Cyclin D1 and CDK4 were observed in group HG and group C, while TRL significantly decreased Cyclin D1 and CDK4 (Fig. 6B, C). These results suggest that HG could promote cell proliferation by speeding up the cell cycle during the first 48 h, but TRL could block the HG-induced cell cycle in the G1/S-phase.

### TRL reduced HMCs apoptosis via the Wnt/β-catenin pathway

**Fig. 7:**
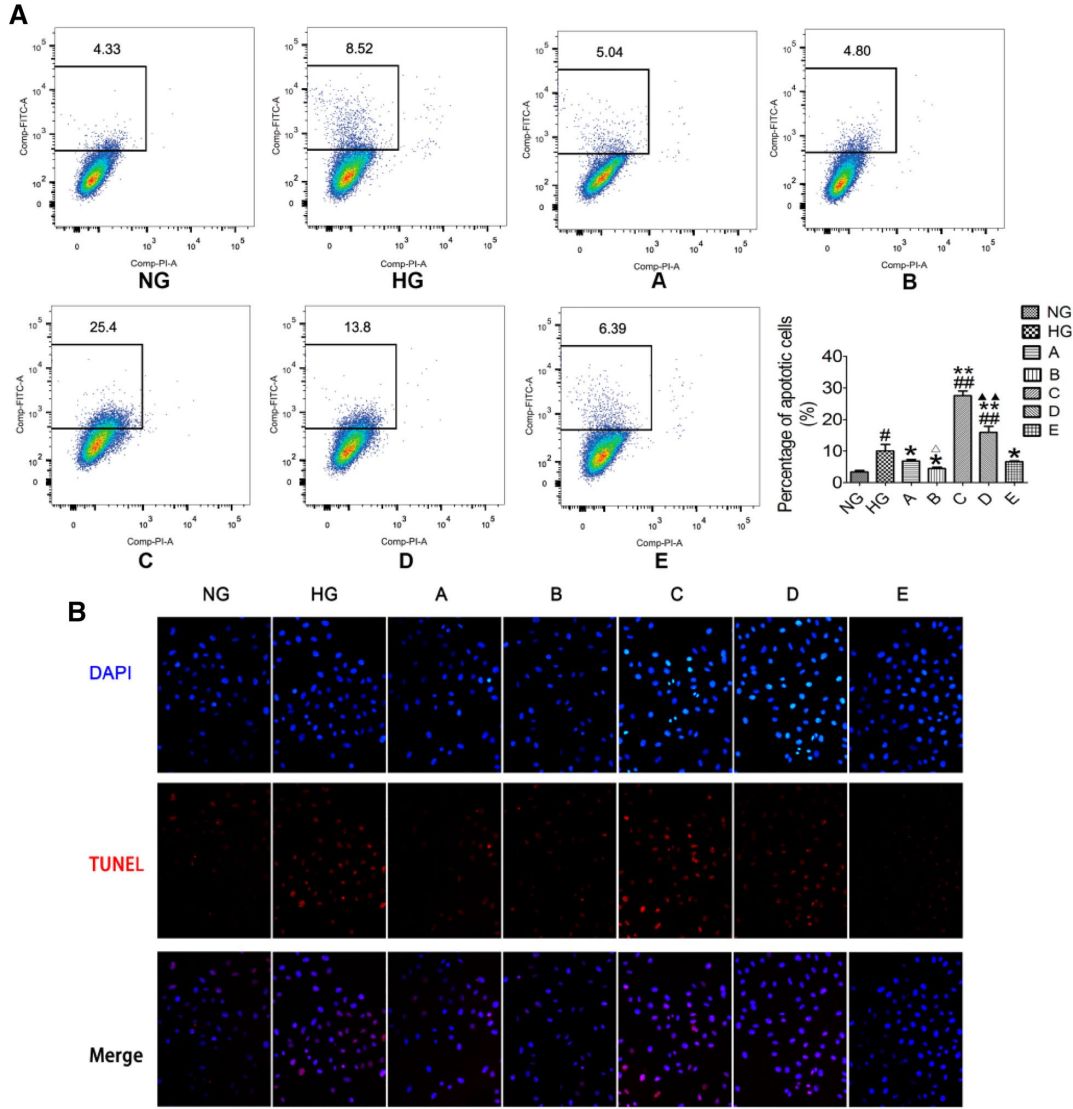
The effect of TRL on apoptosis in HMCs. **A** Representative flow cytometry graphs are shown. Early apoptotic cells are located in the grid, and the numbers above them represent the percentage of apoptotic cells. **B** DNA fragmentation in HMCs detected by TUNEL (×200). Cells that were positive for terminal deoxynucleotidyl transferase-mediated deoxyuridine triphosphate-biotin nick end-labeling (TUNEL) exhibited red staining in the nucleus. DAPI solution was used to stain nuclei (blue). *NG* normal glucose, *HG* high glucose, **A** TRL + high glucose, **B** TRL + β-catenin siRNA + high glucose, **C** β-catenin pcDNA + high glucose, **D** TRL + β-catenin pcDNA + high glucose, **E** ICG-001 + high glucose; ^##^P < 0.01 versus NG; *P < 0.05 versus HG, **P < 0.01 versus HG, ^△^P < 0.05 versus A; ^▲▲^P < 0.01 versus C

As shown in Fig. 7A, the HG group's apoptosis rate was significantly increased (P < 0.05). The apoptosis rate increased significantly following transfection with β-catenin pcDNA (P < 0.01). TRL could reduce the apoptosis induced by β-catenin pcDNA. The rate of early apoptosis observed following the treatment with TRL alone (group A) and in combination with the silencing of β-catenin (group B) was significantly lower than that of group HG (P < 0.05). There was a significant difference between groups B and A (P < 0.05). The apoptosis of HMCs after ICG-001 intervention (group E) also decreased significantly (P < 0.05). This is consistent with the results of the TUNEL assay (Fig. 7B). TUNEL staining indicated that HG and the overexpression of β-catenin induced DNA fragmentation, a sign of apoptosis. Under HG conditions, HMCs displayed DNA fragmentation. However, only a few cells treated with TRL showed DNA fragmentation. DAPI staining showed that some nuclei had a rounded shape.

Interestingly, group C had more DNA fragmentation than the HG group (Fig. 7B). This difference may arise from the differential expression of β-catenin. Both results indicated that HG could lead to cell apoptosis after 72 h. The presentation of β-catenin might be related to the apoptosis of HMCs; its accumulation in the cytoplasm may lead to apoptosis to a certain extent. However, TRL and the regulation of β-catenin could be attributed to the reduction of cell apoptosis.

## Discussion

DN is a glomerular sclerosis disease caused by the abnormal metabolism of diabetes mellitus. High glucose quickly affects mesangial cells, which in turn cause cellular stress and damage [20]. The proliferation and hypertrophy of mesangial cells and the accumulation of an extracellular matrix eventually led to glomerular sclerosis. HG was recognized as the main characteristic of DN and as a risk factor for the occurrence and development of DN [21]. Research confirmed that HG could promote the proliferation of HMCs [22–24]. Our study shows that TRL can inhibit HG-induced HMC over-proliferation in a concentration-dependent manner. HG causes mesangial cell damage because HG activates a molecular pathway leading to apoptosis. HG reduces the survival rate of glomerular cells in various ways, such as increasing oxidative stress or altering growth factor expression [25, 26]. To our knowledge, the biological effects of TRL in measuring the survival of HMCs under HG conditions are not clearly defined. Therefore, TRL’s effect in inhibiting the proliferation of HMCs might become an effective treatment for the prevention and treatment.

The development of DN is associated with the abnormal proliferation of mesangial cells, and HG conditions could induce the proliferation of mesangial cells. In this experiment, the effect of TRL on the expansion of HMCs was studied by MTT assays. We found that the absorbance increased significantly in the HG group at 48 h, suggesting that hyperglycemia stimulated excessive proliferation of HMCs within the experimental dose range. TRL could inhibit the HMCs hyperproliferation induced by HG in a concentration-dependent manner. After the HG treated cells were cultured for 72 h, cell proliferation slowed and cell viability significantly decreased. However, TRL could enhance HMC activity at this time. Since HG conditions had the most significant effect on the addition of HMCs at 48 h, this time point was selected to study further the effect of TRL on the proliferation of HMCs under HG and understand its mechanism.

There are three phases of the cell cycle: G0/G1 phase, DNA synthesis-related S phase, and G2/M phase [27, 28]. Cyclin D1 is a cell cycle regulator of CDK4, and it acts mainly in the G1 phase. The active complexes formed by their combination can promote cells to enter the S phase from the G1 phase. The S phase is the DNA replication phase. Once the cells enter the S phase, cell division can proceed until the subsequent G1 phase. The HMCs proliferated at low rates under normal glucose conditions and largely remained in the G0/G1 phase of the cell cycle. Since HG stimulation, cells in the G0/G1 phase are reduced, increasing the S and G2/M phases. TRL could significantly reverse these effects. These results demonstrate that HG mainly played a role in promoting proliferation. Still, TRL might reduce the HG-induced over-proliferation of HMCs by inhibiting their transition from the G0/G1 to S phase. To explore whether there is a relationship between cell proliferation and the Wnt/β-catenin pathway, we transiently over-expressed β-catenin. We found that the cell cycle in group C speeded up significantly, whereas group D, in which TRL slowed, had the opposite effect. Cyclin D1 and CDK4 was increased in group HG and group C, while TRL significantly decreased their expression. The results above concluded that HG conditions and high expression of β-catenin could accelerate the cell cycle and promote cell proliferation. At the same time, TRL could down-regulate the expression of Cyclin D1 and CDK4 and regulate G1/S phase monitoring to cause G1 arrest. Thus, TRL could slow down the cell cycle. TRL and the Wnt/β-catenin pathways might participate in the cell cycle process.

The Wnt/β-catenin signaling pathway plays an important role in the pathogenesis of DN. Furthermore, numerous studies demonstrated that specific blockade of the Wnt/β-catenin pathway prevented DN progression, including abolishing cell proliferation and ECM expression in glomerular HMCs [29, 30]. The Wnt family comprises of 19 different Wnt ligands. A previous study demonstrated that several canonical Wnts, including Wnt 1, 3, 4 and 5a, promoted the development of kidney disease, such as diabetic kidney disease. Thus, blocking the activation of the Wnt/β-catenin signaling pathway is an important strategy for preventing DN. In the present study, we used pcDNA3.1-β-catenin and siRNA transfected cells to overexpress or knockdown β-catenin. Then we detected the expression levels of β-catenin, TCF4, Wnt5a, Wnt4, and GSK-3β by western blotting and qRT-PCR. After transient transfection of plasmids into HMCs, the signal intensity of the Wnt/β-catenin signaling pathway significantly increased. Still, the expression of related factors in the Wnt/β-catenin signaling pathway was reduced considerably after the 100 μM TRL intervention. These results demonstrate that HG conditions can activate the Wnt/β-catenin signaling pathway, leading to an increase in β-catenin expression. Additionally, TRL could down-regulate the face of β-catenin mRNA and protein levels and inhibit the Wnt/β-catenin pathway. The intervention with TRL alone significantly reduced the expression of β-catenin, TCF4, Wnt5a, Wnt4, and GSK-3β. The expression of these factors was also reduced after the knockdown of β-catenin and the TRL intervention at the same time. This suggests that TRL not only down-regulates the high expression of β-catenin but also regulates other factors in the Wnt/β-catenin signaling pathway.

Wnt/β-catenin signaling is required to protect glomerular mesangial cells from high glucose-mediated apoptosis [31–33]. β-Catenin signaling is involved in many cell processes, for example, cell proliferation, differentiation, and apoptosis. We recently examined the effect of TRL on the sustained effect of Wnt/β-catenin signaling and its effect on reducing apoptosis in HG-stressed mesangial cells. We found that the percentage of apoptotic cells slightly increased under HG conditions after 72 h. The apoptosis rate significantly increased in group C, in which β-catenin was overexpressed. This implies that the high expression of β-catenin might lead to cell apoptosis. These results imply that apoptosis-related molecules are modulated by regulating the canonical Wnt/β-catenin signaling pathway caused by HG induction of HMCs apoptosis. The knock-down in β-catenin levels following treatment of HMCs with TRL might account for the survival of HG-stressed cells.

In short, results demonstrated that HG condition and β-catenin overexpression could activate the Wnt/β-catenin signaling pathway. At the same time, TRL inhibited Wnt/β-catenin signaling by regulating the expression of β-catenin, TCF4, and other related factors. TRL could down-regulate the expression of Cyclin D1 and CDK4, stopping the cell cycle at the G1 phase, inhibiting abnormal proliferation is induced by HG and over-expression of β-catenin. TRL inhibited apoptosis by down-regulating β-catenin signaling. This provides a novel insight into understanding the molecular and signaling pathways through which TRL suppresses HG-induced cell over-proliferation and apoptosis. Thus, treatment with TRL and the modulation of the Wnt/β-catenin pathway represent novel therapeutic strategies for the treatment of DN in the clinical setting.

## Data Availability

The data used to support the findings of this study are included within the article.
